# The use of hormonal contraceptives in high performance Brazilian female swimmers: a cross-sectional study

**DOI:** 10.61622/rbgo/2025rbgo82

**Published:** 2025-10-21

**Authors:** Ana Carla Carvalho, Natani Gaio, Patrícia Cristiane Müller, Simone Carla Benincá

**Affiliations:** 1 Campo Real University Center Guarapuava PR Brazil Campo Real University Center, Guarapuava, PR, Brazil.

**Keywords:** Contraception, Hormonal contraceptive, Swimming athletes

## Abstract

**Objective:**

Women may experience cognitive, emotional, and physical changes during the menstrual cycle, which can impact their physical and athletic performance. This study aimed to investigate whether Brazilian female swimmers in the senior category, i.e., over 18 years of age, federated with the Brazilian Confederation of Aquatic Sports (CBDA), use hormonal contraceptives (HCs) and assess the impacts on their health, athletic performance, and overall well-being.

**Methods:**

This is a cross-sectional descriptive observational study conducted via an online questionnaire based on the Google Forms® platform, sent via WhatsApp® and email to the participants. Questions were asked about age, use of contraceptive methods, observed changes in performance, among others. A total of 136 senior athletes participated in the study. Inclusion criteria were senior female athletes using hormonal contraceptive methods. Athletes who were not in the senior category and/or did not use hormonal contraceptive methods were excluded from the research.

**Results:**

A total of 136 senior athletes responded to the questionnaire. Of this total, 108 reported using hormonal contraceptive methods and were therefore included in the study. The results indicate that the majority of participants use oral contraceptives, with 61 (57%) using this method. Regarding the reason for using hormonal contraceptives, 88 (82.4%) use the method to control the menstrual cycle, in addition to alleviating menstrual symptoms, 63 (59.2%); 62 (57.9%) reported improvement in mood patterns, and 69 (64.55%) stated that they experience benefits from using the method.

**Conclusion:**

It is concluded that the use of hormonal contraceptives presents benefits related to the professional careers of athletes. Besides improving their training performance, HCs also help alleviate menstrual symptoms and control the menstrual cycle and flow.

## Introduction

Female participation in sports has been increasing considerably in recent years, and swimming stands out as one of the sports that has received more attention and recognition. In this context, with the growing female presence in swimming pools, there arises the need to address specific issues related to the health and well-being of these athletes, including the use of hormonal contraception methods.

Throughout the menstrual cycle, the concentrations of different hormones can vary, particularly estrogen and progesterone. These fluctuations during the cycle often negatively affect women's daily lives, at school, at work, and in sports practice.^(1)^

The use of contraceptive methods among female swimming athletes has raised important and complex questions regarding both their impact on sports performance and potential side effects and health risks. Many women experience premenstrual and menstrual symptoms such as cramps, increased flow, loss of concentration, emotional lability, and loss of motivation, which can affect different aspects of training and competition, harming personal life and especially sports performance. This fact appears to be particularly worse among non-users of hormonal contraceptives.^(2)^

Hormonal contraceptives (HCs) are exogenous hormones that inhibit ovulation, preventing pregnancy, but they can also be used to reduce the negative symptoms associated with the menstrual cycle by acting on physiological hormonal fluctuations, inhibiting ovulation, and reducing endogenous secretion levels.^(1)^ Therefore, it is believed that oral contraceptives are the most used method by high-performance Brazilian female swimming athletes to reduce the negative symptoms associated with the menstrual cycle, which can affect performance in training and competitions.

Thus, the study is relevant as it contributes to a broader reflection on the topic, offering important information for athletes, health professionals, and sports managers to promote a responsible and well-founded approach to the use of hormonal contraception in female swimming athletes, ensuring health protection and promoting a successful and healthy sports career.

Therefore, this study aims to investigate the use of hormonal contraception methods among female swimming athletes, considering their impacts on health, sports performance, and the general well-being of these athletes.

## Methods

This research is an individual descriptive analytical observational study of a cross-sectional type. It was conducted remotely using Google Forms® from January to October 2023. To obtain the personal contact information of the athletes, the CBDA was asked for the emails and phone numbers of the female coaches from the major swimming clubs in Brazil. The form was sent via WhatsApp® and email to the athletes registered with CBDA.

The form included an Informed Consent Form (ICF) and a questionnaire developed by the researchers containing 10 closed-ended questions. Participants only had access to the questions after they had provided their consent and accepted the ICF.

A total of 136 female senior athletes, i.e., over 18 years of age, registered with the CBDA, participated in the study. Inclusion criteria were: female athletes in the senior category, which includes women over 18 years of age, registered with the CBDA, who use hormonal contraceptive methods containing estrogen, progesterone, or both. These methods include oral contraceptive pills, intrauterine systems (IUS), hormonal implants, injectable contraceptives, vaginal rings, or transdermal patches. Participants also had to agree to the study protocol and sign the Informed Consent Form (ICF). Exclusion criteria included female athletes who did not fall into the senior category (i.e., women under 18 years of age or over 40 years old), those who did not use hormonal contraceptive methods, or those who did not agree to the study protocol or did not sign the ICF.

The questions included in the form were about age, use of contraceptive methods, which method is used, the hormonal composition of the method, duration of use, motivation for use, changes in body fat percentage, mood patterns, and observed changes in performance.

The variables analyzed were age, use of hormonal contraceptives, the type of contraceptive used, reasons for using the contraceptive, presence of benefits or drawbacks after starting contraceptive use, and any improvement or deterioration in athletic performance with the use of the contraceptive method. Among these variables, the participants’ age and duration of contraceptive use were discrete quantitative variables, while sex, use of hormonal contraceptives, the method and composition of the contraceptive, and the analysis of benefits or drawbacks related to hormonal contraceptive use and its impact on athletic performance were nominal qualitative variables.

Regarding the variables, the following were highlighted: age of the participants and duration of contraceptive use were discrete quantitative variables. On the other hand, the nominal qualitative variables included: gender (female), use or non-use of hormonal contraceptives, the method and composition of the contraceptive, and the analysis of benefits or drawbacks of hormonal contraceptive use in relation to athletic performance.

The data were tabulated using Microsoft Office Excel® and, after tabulation, analyzed with the help of the Statistical Package for the Social Sciences (SPSS®), version v26.0. The Chi-square test was used to compare the nominal qualitative variables. A significance level of p ≤ 0.05 was adopted.

This research was approved by the Research Ethics Committee of Centro Universitário Campo Real (CEP), under opinion number 5.832.806/2022 of December 21, 2022, Certificado de Apresentação de Apreciação Ética number 65630622.6.0000.8947 in accordance with the ethical considerations outlined in Resolution CNS 196/96, updated by 466/2012 or 510/2016, and conducted in accordance with the Declaration of Helsinki.

## Results

**Figure 1:**
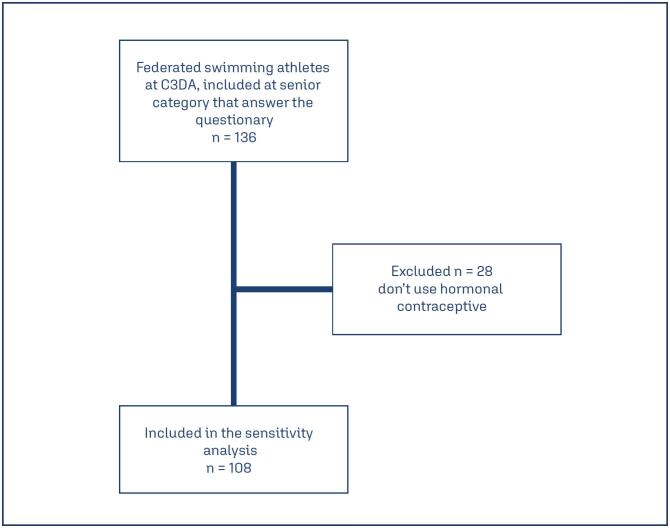
Patients selection process

In the present study, 136 forms filled out by female athletes, federated with CBDA and classified in the senior category, were analyzed. Of these, 28 were excluded because they reported not using hormonal contraceptives. (Figure 1)

The prevalence of hormonal contraceptive use was 87.7%. The methods reported were: 39.9% used an IUD (Mirena® or Kylena®), 3.7% used an intradermal implant, and 57% used the pill. Regarding the type of hormone present in the contraceptives, 42.1% reported using combined hormones (estrogen + progesterone), 26.2% used only progesterone, and 31.8% did not know the information. Concerning the duration of contraceptive use, 19.6% have used the method for less than a year, 32.7% for one to two years, and 47.7% for more than two years (Table 1).

**Table 1:** Description of the sample and characteristics related to contraceptive use

Variables		n(%)
Age		
	18 - 25	84(61.8)
	26 - 30	41(30.1)
	31 - 35	10(7.4)
	36 - 40	1(0.7)
Use some contraceptive		
	Yes	109(80.1)
	No	27(19.9)
Use some hormonal contraceptive		
	Yes	107(78.7)
	No	29(21.3)
Kind of hormonal contraceptive in use		
	Hormonal IUD (Mirena®, Kylena®)	42(39.9)
	Intra-dermal implant	4(3.7)
	Pill	61(57.0)
Which hormone		
	Progesterone	28(26.2)
	Estrogen and Progesterone	45(42.1)
	Do not know	34(31.8)
Duration of use		
	1 - 2 years	35(32.7)
	> 2 years	51(47.7)
	> 1 year	21(19.6)

Regarding the performance of athletes using hormonal contraceptives, 64.55% reported experiencing benefits from their use, 19.6% noted adverse effects, and 15.9% reported no noticeable difference. Concerning body fat percentage, 30.8% reported no increase, while 50.5% reported an increase. Among athletes using hormonal contraceptives, 57.9% observed an improvement in mood patterns. The questionnaire also addressed events occurring after the initiation of hormonal contraceptive use. When evaluating these events, 28.7% of participants reported improved training performance, in contrast to 22.2% who experienced difficulty gaining muscle mass, 11.1% who noted a decrease in strength, and 7.4% who reported worsened training performance (Table 2).

**Table 2:** Characteristics of contraceptive use reported by swimming athletes of the Brazilian Federation

Variables		n(%)
Change of athletics performance after starting the use of hormonal contraceptives		
	Benefits from the use	69(64.5)
	Harms from the use	
Increase in body fat percentage after starting the use of hormonal contraceptive		
	No	33(30.8)
	Yes	54(50.5)
	Do not know	20(18.7)
Improvement in mood after starting the use of hormonal contraceptive		
	Yes	62(57.9)
	No	45(42.1)
Changes observed after starting the use of hormonal contraceptive		
	Improvement in training performance	31(28.7)
	Difficulty gaining muscle mass	24(22.2)
	Decrease in strength	12(11.1)
	Worsening of training performance	8(7.4)
	None of the options	38(5.1)

**Figure 2:**
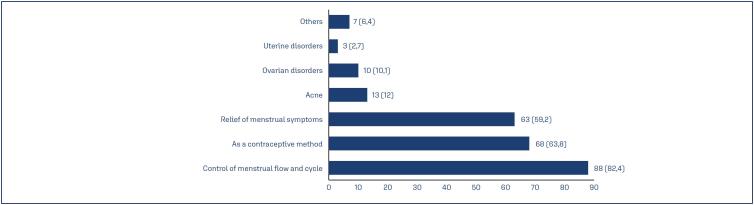
Reasons for using contraceptives

When asked about the reasons for using hormonal contraceptives, with the option to select more than one reason, the main reasons were control of menstrual flow and cycle (82.4%), use as a contraceptive method (63.8%), and relief from menstrual symptoms (59.2%) (Figure 2).

## Discussion

The results showed that more than half of the athletes participating in this study are aged between 18 and 25 years, with a prevalence of CH use at 87.7%, and the pill being the most cited. 68.3% of these athletes reported knowing which hormone is present in the CH they consume, and 80.4% have been using it for more than a year.

Regarding the performance of athletes using hormonal contraceptives, 64.55% reported benefits, 19.6% reported adverse effects, and 15.9% noticed no difference.

The physiological menstrual cycle in women is controlled by a complex interaction of hormones, with concentrations varying in different phases of the cycle. This cycle lasts an average of 28 days, when unchanged, and has three phases: follicular, ovulatory, and luteal. In the first phase, progesterone and estrogen levels are low and gradually increase until in the luteal phase, both progesterone and estrogen are at high levels.

These hormonal changes can lead to physical and mental alterations in the body, resulting in Premenstrual Syndrome (PMS), which includes physical, mood, cognitive, and behavioral changes with discomfort, irritability, depression, or fatigue complaints during the period preceding menstruation.

Many women use hormonal methods to relieve symptoms and improve their quality of life. The data obtained in this study showed that about 59.2% of the athletes surveyed suffer from PMS and use hormonal contraceptives to alleviate these symptoms. In a similar study, more than half of the participants suffer from PMS, with symptoms ranging from abdominal pain, lower back pain, cramps, headaches or migraines, decreased concentration, low motivation, anxiety, or irritability.^(3)^

Other studies have shown that female athletes have an increased prevalence of PMS, suggesting that intensive training may influence the occurrence of PMS,^(4)^ as both progesterone and estrogen can interfere with physiological functions such as anaerobic and aerobic capacity, muscle strength, among others.

The use of CHs is an effective strategy to prevent pregnancy, as the pills maintain constant levels of female hormones, thus attenuating the hormonal variations observed during the menstrual cycle.

Pregnancy has a considerable impact on a woman's life. In the sports context this impact intensifies, as the athlete's work is her body, which will inevitably undergo changes during pregnancy. In this study, 63.8% of the swimmer athletes use hormonal contraception solely as a contraceptive method. This is likely due to the various dimensions in which motherhood impacts an athlete's career.

Besides preventing unwanted pregnancy, reducing menstrual symptoms, and regulating the menstrual cycle, CHs also help prevent common diseases in the female reproductive system, such as ovarian cysts, ovarian or endometrial cancer, pelvic inflammatory disease, and benign breast disease. In this study, 12.8% of the athletes reported using CHs as a therapeutic resource for ovarian and/or uterine disorders, proving the importance of this treatment in practice.

Another reason women generally use CHs is to improve acne, a condition that can be treated with CHs. A study comparing the use of CHs with placebos and analyzing acne lesion improvement concluded that there is significant improvement in women using CHs.^(5)^ This focus on medication use is quite common; however, in this study, only 12% of respondents reported using such a contraceptive method to reduce acne.

As mentioned earlier, hormonal contraceptives are effective methods for maintaining constant hormone levels throughout the menstrual cycle. Additionally, CHs are not ergolytic concerning the athlete's routine, as the timing of bleeding can be manipulated, allowing control of the menstrual cycle and flow, making them more regular, predictable, and less intense.

In this study, it was observed that among the reasons why athletes use contraceptives, 82.4% reported that cycle and menstrual flow control was the main reason for starting the medication, to control bleeding during training and competitions.

It is essential to understand the potential risks and benefits of contraceptives. The negative aspect related to CHs mentioned in the literature is weight variation due to increased energy intake in the luteal phase, caused by increased appetite due to hormonal fluctuation.^(6)^ Regarding body fat percentage, 30.8% reported no increase, 50.5% reported an increase, and 18.7% were unsure.

Although most women with regular menstrual cycles experience PMS, not all are affected by mood changes. However, studies strongly indicate that sex hormones directly influence cognition and mood, with estrogen having an antidepressant effect causing positive mood changes, while progesterone is associated with decreased serotonin, leading to a depressive state.^(7)^

In this study, among athletes using hormonal contraceptives, 57.9% noticed an improvement in mood after starting hormonal methods, while 42.1% did not notice this improvement. Given the importance of mood, studies show that having a positive mood before competition is associated with excellent performance during competitions.^(7)^

Regarding training and competition performance, 28.7% of athletes reported improved performance with CH use. This improvement may be because CHs inhibit the physiological hormonal fluctuations of the menstrual cycle.

In another study, Rechichi et al.^(8)^ analyzed the performance of swimming athletes in 200-meter events, noting that although there was no difference in 200-meter times, CH use helps reduce the lactate peak available in the blood, leading to faster muscle recovery by reducing hormonal fluctuations. Only 7.4% reported a decline in training performance after starting CH use, indicating that the use of estrogen and progesterone hormones does not generate significant negative effects on women's physical training performance within a hormonal contraceptive cycle. The low percentage related to the worsening of training found in the present study corroborates with the study by Elliott-Sale et al.^(9)^

Among athletes who reported a decline in training performance with CH use, 22% highlighted the difficulty in gaining muscle mass. Riechman et al. ^(10)^ pointed out that oral contraceptive use might harm muscle gains in young women. Examining the effects of oral CHs on muscle responses in two groups of young, healthy women participating in a standardized resistance exercise training program detected significant differences in lean mass gains between the groups, with decreased plasma concentrations of dehydroepiandrosterone (DHEA), dehydroepiandrosterone sulfate (DHEAS), and insulin-like growth factor 1 (IGF-1), and increased cortisol levels in the CH user group, resulting in significant differences in lean mass gains. It is noted that decreased lean mass gains may be related to CH effects on anabolic and catabolic hormone levels or the androgenicity of the progestin, which can bind to androgen receptors and inhibit their function.

The literature regarding possible influences of CHs on muscle strength and athletic performance presents some divergence. A study by Procter-Gray et al.^(11)^ indicated a positive relationship between oral CH use and improvements in strength and muscle mass, as users obtained greater lean mass gains over the treatment period. The authors attribute the advantage of CH use to the various estrogen receptors present in skeletal muscle, which are anabolically affected by exogenous estrogen. On the other hand, some studies have shown no relationship between CH intake, strength gain, and athletic performance, as revealed in the present study, where only 11.1% of swimming athletes reported decreased strength during CH use.

## Conclusion

Hormonal contraceptives are the most commonly used method among female swimmers and are considered to provide numerous benefits related to their professional careers. Their primary purpose is to control the menstrual cycle and flow, followed by their use as a contraceptive method and to alleviate menstrual symptoms. Additionally, most athletes reported a significant improvement in training performance. Regarding the potential influences of HCs on muscle strength and athletic performance, it is not possible to assert that they have significant effects on muscle strength and athletic performance. Longitudinal studies with this focus are needed to clarify the effects of hormonal contraceptives on swimmers and their potential consequences for performance and body composition. Research on combined oral contraceptives in elite athletes is currently insufficient and may be hindered by inadequate research design and methodology, as well as small sample sizes. Based on this study, it is clear that more randomized clinical trials are needed to evaluate the variety of HC formulations currently available and their effects on the health and performance of elite athletes. Furthermore, it is essential to elucidate the influences of hormonal contraceptives in the sports domain and their consequences on body composition and athletic performance.

## Data availability

: The authors did not make the data from this article available in repositories prior to submission.
